# Complete Genomic Sequences of an H3N8 Equine Influenza Virus Strain Isolated in China

**DOI:** 10.1128/genomeA.00654-13

**Published:** 2013-08-22

**Authors:** Chao Zhu, Qian Li, Wei Guo, Gang Lu, Xin Yin, Ting Qi, Wenhua Xiang, Duoliang Ran, Juanjuan Qu

**Affiliations:** College of Life Sciences, Northeast Agricultural University, Harbin, People’s Republic of Chinaa; Harbin Veterinary Research Institute of the Chinese Academy of Agricultural Sciences, Harbin, People’s Republic of Chinab; College of Veterinary Medicine, Northeast Agricultural University, Harbin, People’s Republic of Chinac; College of Animal Science, Xinjiang Agricultural University, Urumchi, People’s Republic of Chinad

## Abstract

We report the complete genomic sequence of A/equine/Heilongjiang/1/2010, a strain of Florida sublineage clade 2 of H3N8 subtype equine influenza virus (EIV) isolated in northern China. This is the first announcement of a complete genomic sequence of EIV of such a clade in China.

## GENOME ANNOUNCEMENT

Equine influenza virus (EIV) belongs to the *Orthomyxoviridae* family and is responsible for the severe acute respiratory disease of equine influenza (EI) in the horse population. Until now, only two subtypes of EIV have been isolated, H7N7 and H3N8. The former is regarded as having been extinct while the latter evolves constantly and has diverged into two antigenically and genetically different evolutionary lineages since 1986, the American and the European lineage. The American lineage further evolves into the Kentucky, South American, and Florida sublineage clades 1 and 2 (1–3). China had experienced three main EI outbreaks before 2007, from which the H7N7, avian-like H3N8, and European-like H3N8 subtype EIVs were isolated, respectively. Between 2007 and 2008, the fourth serious outbreak of EI occurred in China (4–6). The causal antigen was identified as Florida sublineage clade 2 of H3N8 subtype EIV (4–6). However, up to now, only a partial hemagglutinin (HA) sequence of the Chinese EIV isolate of Florida sublineage clade 2 has been published, and the complete genome sequence of a virus of this clade has not been reported.

One restricted outbreak of EIV was observed in Heilongjiang Province, China, in 2010. The causal virus, named A/equine/Heilongjiang/1/2010, was confirmed to be a strain of Florida sublineage clade 2 of H3N8 EIV after sequencing. The employed primers in the study were designed using Oligo (version 6.0). The eight fragments were amplified separately by standard reverse transcription (RT)-PCR and sequenced by the Invitrogen Corporation (Shanghai, China). The sequencing results revealed that the genome of A/equine/Heilongjiang/1/2010 was composed of eight fragments, PB2, PB1, PA, HA, NP, NA, M, and NS, whose full lengths were 2,280, 2,274, 2,151, 1,704, 1,497, 1,413, 982, and 838 nucleotides, respectively. Aligned with the conserved gene, no deletions or insertion mutations were found. When we compared the sequence data with other published sequences of EIV, the levels of identity with most of circulating Florida-2 strains were more than 99% in each segment, which was also proven by phylogenetic analysis. Analysis of the deduced antigenic sites in the HA of A/equine/Heilongjiang/1/2010 indicated that few amino acid substitutions existed compared with those of circulating Florida-2 strains. However, the actual antigenic differences cannot be estimated by the deduced amino acid sequences alone, and further research is needed.

Our study revealed the complete genomic sequence of a virus strain of the Florida sublineage clade 2 of the H3N8 subtype EIV, A/equine/Heilongjiang/1/2010. It is the first announcement of such a clade of EIV in China. Our study is meaningful for furthering understanding of the molecular evolution of H3N8 EIV and will facilitate future investigations of the epidemiology of these viruses.

### Nucleotide sequence accession numbers. 

The genome sequences of A/equine/Heilongjiang/1/2010 have been deposited in GenBank under the accession numbers KF309031 to KF309037 (PB2, PB1, PA, NP, NA, M, and NS) and accession number JQ265982 (HA).
